# Pan-Amyloid Reactive Peptides p5+14 and p5R Exhibit Specific Charge-Dependent Binding to Glycosaminoglycans

**DOI:** 10.3390/ph18091340

**Published:** 2025-09-06

**Authors:** Trevor J. Hancock, Angela D. Williams, James S. Foster, Jonathan S. Wall, Emily B. Martin

**Affiliations:** Department of Medicine, The University of Tennessee Health Science Center College of Medicine, Knoxville, TN, 37920, USA; thancock1@utmck.edu (T.J.H.); awilliam@utmck.edu (A.D.W.); jfoster3@uthsc.edu (J.S.F.); jwall@utmck.edu (J.S.W.)

**Keywords:** amyloidosis, peptide p5+14, peptide p5R, heparin binding peptides, PET imaging, diagnostics, pan-amyloid peptides

## Abstract

**Background**: Polybasic peptides are being developed as components of reagents for diagnosing and treating patients with systemic amyloidosis. In addition to fibrils, amyloid deposits ubiquitously contain heparan sulfate proteoglycans. We have hypothesized that pan amyloid-targeting peptides can specifically engage, in addition to fibrils, a subset of glycosaminoglycans (GAGs) with high negative charge density. In this study, we characterized the binding of peptides p5+14 (a PET imaging agent for amyloid [^124^I-evuzamitide]) and p5R (a fusion protein used in the therapeutic AT-02) to GAGs. **Methods**: The peptide structure was evaluated in the presence of low molecular weight heparin using circular dichroism, and their interaction with synthetic GAGs of varying length and charge was interrogated. The binding patterns of p5+14 and p5R were compared using correlation analyses. **Results**: The peptides exist as mixed structural-fractions in solution but adopt an α-helical structure in the presence of heparin. Both peptides preferentially recognize heparin and heparan sulfate GAGs with a linear positive correlation between binding and the total charge and charge density. **Conclusions**: These peptides have previously been shown to specifically target amyloid deposits in vivo. A component of this specificity is their preferential interaction with a subset of heparan sulfate GAGs that have high charge density, potentially related to the degree of 6-O-sulfation. These data support the hypotheses that amyloid-associated GAGs have unique sulfation patterns, thereby explaining why these peptides do not bind GAGs found on the plasma membrane and extracellular matrix of healthy tissues.

## 1. Introduction

The systemic amyloidoses comprise a group of protein misfolding disorders characterized by the extracellular deposition of protein in visceral organs and tissues. Over 30 proteins have been shown to form pathologic amyloid deposits in humans [1,2]. At least 20 distinct types of systemic amyloidosis have been identified, each characterized by the specific protein that comprises the amyloid fibril [1,2,3]. The most prevalent types of systemic amyloidoses are associated with the deposition of fibrils composed of transthyretin, either wild type (ATTRwt) or variant (ATTRv) proteins, or monoclonal immunoglobulin light chains (AL amyloidosis). The composition of tissue amyloid deposits—the amyloid proteome—is complex. In addition to amyloid fibrils, the deposits contain non-fibrillar, accessory proteins including extracellular matrix components (e.g., collagen and heparan sulfate proteoglycans [HSPG]) and serum proteins sequestered from the circulation (notably, apolipoproteins E and A-4, and serum amyloid P component [SAP]) [4,5].

The presence of apolipoproteins, SAP, and HSPG in amyloid deposits is universal. Indeed, these proteins contribute to the amyloid signature seen during the mass spectrometric characterization of tissue amyloid [5]. The ubiquitous presence of glycosaminoglycans in amyloid, generally in the form of the perlecan HPSG, provides the rational for the term *amyloid,* which was given to the lardaceous deposits seen in tissues by early pathologists—amyloid, from the Latin *amylum*, meaning starch-like.

The impact of accessory proteins in amyloid deposits remains unclear; however, some components have been evaluated extensively. Serum amyloid P component, for example, was thought to stabilize the amyloid pathology and render it resistant to proteolysis and immunological clearance [6]. This hypothesis was tested using a small molecule drug (miridesap) that crosslinks SAP, thereby clearing it from amyloid deposits, potentially leading to their clearance [7]. However, in clinical studies (NCT02603172), miridesap failed to promote the meaningful removal of amyloid in patients [8]. This was one of the first clinical studies to assess a therapeutic based on the structural elements of amyloid deposits.

The amyloid-associated collagen, which is intimately associated with the fibrils in the form of helical superstructures [9], may shield amyloid from recognition by macrophages, thereby preventing clearance by the innate immune system [10]. This observation may partly explain why systemic tissue amyloid, non-native aggregates of misfolded protein, is immunologically inert.

Glycosaminoglycans (GAGs) are ubiquitously associated with extracellular amyloid deposits. They have been shown to be important in amyloid formation by providing microenvironments that bind and concentrate amyloidogenic precursor proteins [11,12,13,14,15,16] and/or by lowering the thermodynamic/kinetic barriers that disfavor fibrillogenesis [11,12,13,14,15,16,17]. Heparan sulfate (HS) is the major glycan in amyloid and is presumably contributed principally by cells adjacent to the sites of fibril formation [18]. Moreover, genetic knockout of enzymes required for HS biosynthesis in mice render them resistant to the serum amyloid protein A (AA amyloid) deposition under conditions that favor the pathology [19]. Recent molecular dynamic simulations suggest that heparin, and potentially the S-domains of HS, bind directly with mature amyloid fibrils via multivalent electrostatic interactions involving the sulfate moieties of the GAG and the uncompensated arrays of basic amino acid side chains aligned on the long axis of the fibril [20]. The HS in amyloid is biochemically distinct from that found in the extracellular matrix or present on the plasma membrane as HSPGs of normal tissues [19,21,22]. The HS associated with amyloid is highly sulfated or has a higher density of highly sulfated, heparin-like, S-domains that are trisulfated disaccharide units comprising iduronic acid (2S) and glucosamine (NS, 6S) [22].

Thus, amyloid is a complex heterogeneous matrix that affords multiple structural components that can be targeted during the development of novel amyloid-binding therapeutic and diagnostic reagents. On a macroscopic scale, amyloid fibrils exhibit a remarkably similar structure, regardless of the precursor proteins from which they are formed. The common structure offers promise for the development of pan-amyloid reactive reagents. Alternatively, amyloid fibril-specific antibodies that are based on the recognition of specific neo- or cryptic epitopes (cryptotopes) that form during the misfolding of amyloid precursor proteins as they are incorporated into the amyloid fibril can be generated. This is the basis for many of the ATTR and AL amyloid binding antibodies currently in clinical evaluation [23].

Despite recent advances in the treatment of ATTR and AL amyloidosis, one major goal remains—the effective clearance of tissue amyloid from organs, which may foster the recovery of organ function and improvement in patient outcomes and quality of life. For patients with ATTR amyloidosis, the newly developed treatments focus on slowing amyloid deposition. Stabilizing the tetrameric native state of transthyretin using small molecules (e.g., tafamidis and acoramidis) [24,25] or reducing protein translation using small interfering RNA (e.g., patisiran and vutrisiran) [26,27] or antisense oligonucleotides (e.g., eplontersen) [28] have been transformative for patients. Moreover, there are now three amyloid fibril-binding antibodies being clinically evaluated for their ability to clear tissue amyloid in patients with ATTR or AL amyloidosis (NCT04360434, NCT03336580, NCT04512235, NCT04504825) [29,30,31,32].

With the advent of these new and effective therapeutics, the need to rapidly and accurately diagnose patients with systemic amyloidosis, and notably cardiac amyloidosis, has become paramount. Diagnosing patients with amyloidosis is a continuing challenge with more than 30% of patients experiencing delayed time from symptom onset to definitive diagnosis of more than one year. Many patients see more than four physicians before finally being diagnosed correctly [33,34]. Unfortunately, it is not uncommon for individuals with rare diseases to undergo what has been termed a diagnostic odyssey as they search for clinical answers [34]. This presents a challenge for physicians and patients, as survival and quality of life correlate significantly with early diagnosis, and many therapies are not effective in patients with advanced disease (e.g., tafamidis) [35].

Delayed diagnosis is multifactorial including: physician awareness; difficulty of differential diagnosis; multi-system/organ involvement (heterogenous presentation); complex diagnostic algorithms; and the lack of amyloid-specific, non-invasive, diagnostic tests that can be used to determine the presence or absence of amyloid (of any type in any organ). To address this, we have explored the use of synthetic, polybasic peptides as pan-amyloid targeting agents [36,37,38,39,40,41]. When radiolabeled with nuclides suitable for imaging, these peptides can be used to detect systemic amyloid in preclinical and clinical studies [37,39,40,41,42,43,44,45,46,47,48,49,50,51]. The synthetic peptides p5+14 (which forms the basis of the PET/CT imaging agent iodine *^124^I-evuzamitide* [NCT06788535]) and p5R (a constituent of *zamubafusp alpha*, currently in clinical trial for treatment of cardiac amyloidosis [NCT05951049 and NCT05521022]) form alpha-helices in the presence of ligands and have been developed as pan-amyloid-reactive peptides [40,52].

Both peptides consist of a repeating heptad structure with periodic lysine (p5+14; -XXKXXXK-) or arginine (p5R; -XXRXXXR-) amino acids. Due to their polybasic structure, they undergo electrostatic interactions with molecules expressing linear arrays of high negative charge density—notably amyloid-like fibrils [20,40,52]. These peptides also avidly bind other highly negatively charged linear molecules such as heparin and hypersulfated HSPG, the latter of which is associated with amyloid deposits in vivo [18,20,21,22]. Notably, these peptides do not bind the ubiquitous lower sulfated HSPG present in normal tissues, and consequently, the in vivo biodistribution of ^125^I-labeled p5R differed dramatically from that of the quintessential HSPG binding protein, basic fibroblast growth factor [53]. Because the peptides act as “pattern recognition receptors” for highly electronegative arrays in amyloid, rather than specific amino acid sequences, they exhibit pan-amyloid reactivity. Iodine-124 (^124^I) evuzamitide (^124^I-labeled p5+14) has been shown to bind many distinct types of systemic amyloid by PET/CT imaging including patients with ALκ, ALλ, ATTRv, ATTRwt, ALECT2, AGel, AApoAI, and ALys [49]. Additional clinical studies have demonstrated that ^124^I-evuzamitide can detect early amyloid deposits and yields highly sensitive and specific detection of cardiac amyloid; moreover, the quantitative uptake of radiolabeled peptide correlates significantly with other measures of cardiac structure and function in patients with amyloidosis [44,49].

While we have previously shown that our peptides bind heparin in vitro and accumulate selectively in amyloid-laden organs in mice and humans, there are other potentially reactive GAGs throughout the body (e.g., heparin, chondroitin sulfate [CS], dermatan sulfate [DS], hyaluronic acid [HA], and keratin sulfate [KS]) that polybasic peptides such as p5+14 and p5R may bind. Herein, we have used high-throughput screening of negatively charged, sulfated glycosaminoglycans and heparan sulfate arrays to assess the binding selectivity of peptides p5+14 and p5R, which could contribute to the specific binding to amyloid in vivo. We found preferential reactivity of the peptides with highly sulfated GAGs—predominantly heparin and HSPG. Moreover, the peptides exhibited preferential binding to HS with a high negative charge density.

## 2. Results

### 2.1. Structural Models of Peptides p5+14 and p5R

**Figure 1:**
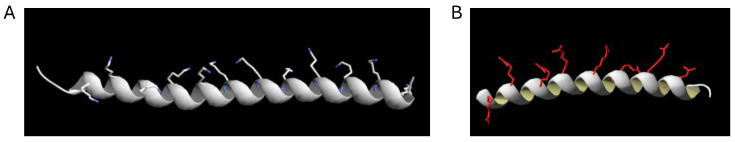
The polybasic peptides p5+14 and p5R are predicted to form α-helices. Ribbon diagrams of peptides p5+14 (**A**) and p5R (**B**) were generated by iTasser and visualized in Deepview/Swiss-PdbViewer v. 4.1.1 (Swiss Institute of Bioinformatics). Positively charged lysine (blue) or arginine (red) side chains are highlighted.

**Figure 2:**
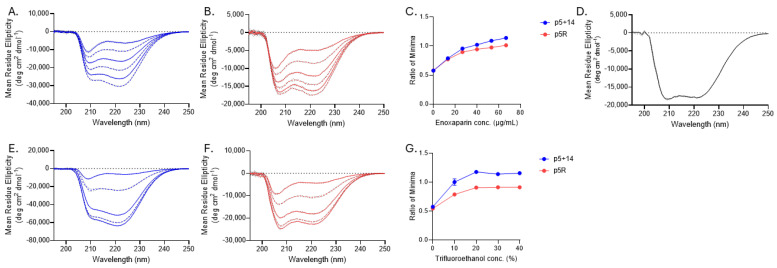
Peptides p5+14 and p5R adopt a helical structure in the presence of low-molecular weight heparin (enoxaparin) or trifluoroethanol (TFE). Secondary structure of the peptides p5+14 (**A**,**E**) and p5R (**B**,**F**) were determined using circular dichroism in the presence of increasing concentrations of enoxaparin (**A**,**B**) or TFE (**E**,**F**). Exemplary spectra of α-helical protein BSA (**D**). The ratio of the two observed minima was determined for each peptide [p5+14 222 nm: 210 nm (blue); p5R 222 nm: 208 nm (red)] with increasing concentrations of enoxaparin (**C**) or TFE (**G**). Representative data with the mean ± SD are shown (*n* = 3).

The cationic peptides p5+14 and p5R consist of heptad amino acid repeats of 45 and 31 amino acids with net positive charges of +12 and +8 for p5+14 and p5R, respectively (Table 1). The N-terminal of each is functionalized to accept radioiodine at the tyrosine residue or chelate a radiometal through coordination at the tri-glycine [50]. Both peptides were designed in silico to form an α-helix with the positively charged lysine (K, p5+14) or arginine (R, p5R) side chains aligned along one face of the helix. This configuration leads to an array of positive charges that can interact with negative charges on the amyloid fibril or amyloid-associated, hypersulfated HSPG (Figure 1) [54,55]. Structural predictions are corroborated by circular dichroism (CD) analyses of protein secondary structure and the change with increasing concentrations of low-molecular weight heparin (enoxaparin) or 2,2,2-trifluoroethanol (TFE) (Figure 2). Initially, CD spectra for both peptides had a large minimum at 208 nm and 210 nm (p5+14 and p5R, respectively) with a smaller minimum at 222 nm (typically corresponding to an α-helix, as demonstrated in Figure 2D). Increasing concentrations of enoxaparin increased the intensity of the two observed minima (Figure 2A,B). At higher concentrations of enoxaparin, the ratio of the minima shifted to 1 or greater (Figure 2C). TFE is a potent inducer of secondary structure by stabilizing intramolecular bonds [56]. When increasing concentrations of TFE are added to p5+14 and p5R, similar changes in the spectra minima were observed (Figure 2E,F). The ratio of the minima also increased to 1 or greater with increasing concentrations of TFE (Figure 2G).

### 2.2. Polybasic Peptides p5+14 and p5R Bind to Systemic Amyloid Deposits

**Figure 3:**
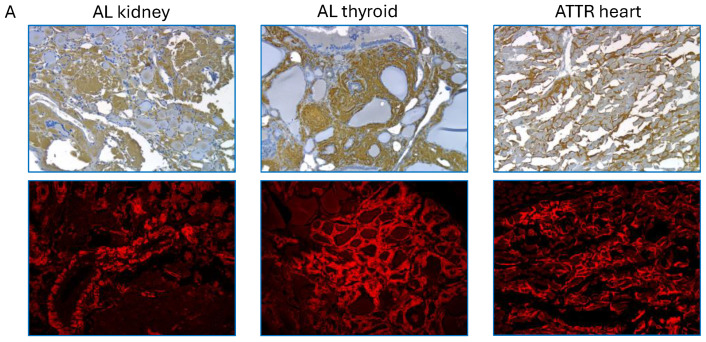

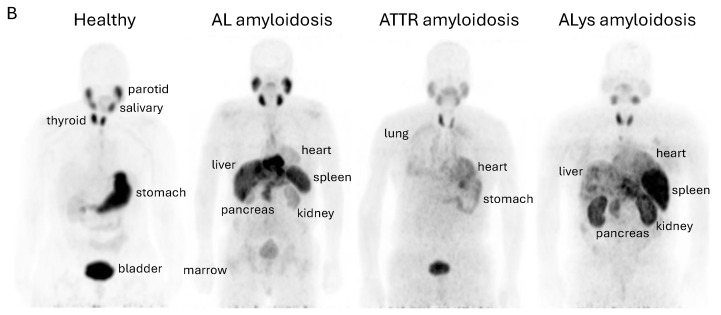
Biotinylated peptide p5R and radiolabeled p5+14 (^124^I-p5+14; ^124^I-evuzamitide) bind specifically to amyloid. (**A**) Biotinylated p5R binding to amyloid deposits in the kidney and thyroid gland of patients with AL amyloidosis and with cardiac amyloid in a patient with ATTR amyloidosis. The distribution of amyloid is shown as red fluorescence in Congo red stained tissue sections. Brightfield and Congo red image magnification was 4× except the ATTR heart, which used a 20× objective. Tissue sections were not consecutive. (**B**) Biodistribution of ^124^I-evuzamitide (^124^I-p5+14) in a healthy subject and patients with AL, ATTR, or lysozyme-associated (ALys) amyloidosis. Maximum intensity projections PET images were acquired at 5 h post-injection of 2 mCi radiotracer.

Peptide p5+14 is the amyloid-targeting domain of both iodine-124 (^124^I-evuzamitide; ^124^I-p5+14) and technetium-99 (^99m^Tc-p5+14) labeled radiotracers, which are in development for the diagnosis of systemic amyloidosis. Peptide p5R represents the amyloid binding domain in the human immunoglobulin-peptide fusion reagent, *zamubafusp alpha* (AT-02), which is currently being clinically evaluated as a treatment for systemic amyloidosis (NCT05951049 and NCT05521022). Both peptides can specifically bind amyloid deposits (Figure 3) [40,49,52,53]. Using immunohistochemistry, we were able to demonstrate the specific association of biotinylated p5R with amyloid deposits in sections of human tissue (Figure 3A). Using PET/CT imaging, the biodistribution of radioiodinated peptide p5+14 in patients with systemic amyloidosis was consistent with uptake in organs and tissues clinically known or suspected of amyloid deposition when compared with healthy subjects where no pathologic uptake was observed (Figure 3B). Notably, ^124^I-p5+14 accumulated within the hearts of patients with AL and ATTR amyloidosis with high sensitivity, supporting the potential of this reagent as a novel diagnostic for cardiac amyloid.

### 2.3. Glycosaminoglycan Binding of Biotinylated Peptides p5+14 and p5R

**Figure 4:**
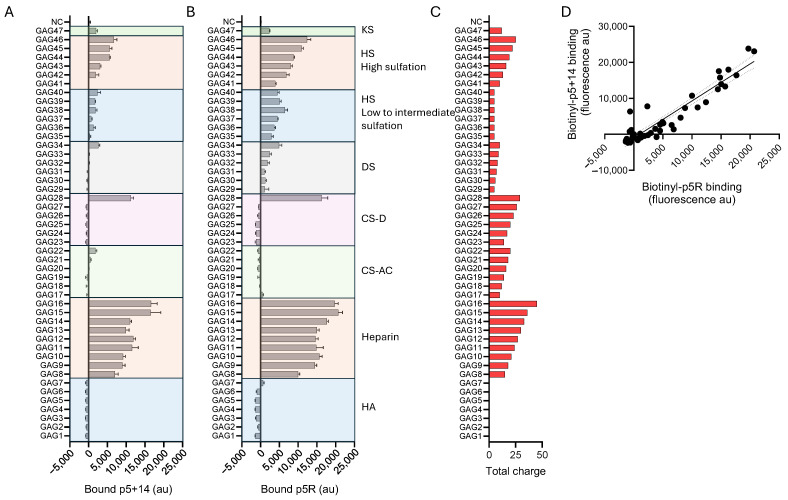
Biotinylated peptides p5+14 and p5R exhibited selective binding to heparin and highly sulfated HS glycosaminoglycans. A GAG array was used to assess the binding of a solution of (**A**) biotinylated-p5+14 and (**B**) and biotinylated-p5R at 2 µg/mL. (**C**) The total charge on each GAG was calculated and plotted for comparison. Binding of each peptide was quantified by measuring the fluorescence emission of a streptavidin fluorophore (fluorescence arbitrary units). (**D**) Pearson’s correlation analysis for p5+14 and p5R binding to GAG was performed and the data fit to a linear regression and plotted with 95% confidence interval. For (**A**,**B**), the mean ± S.D. is shown (*n* = 4).

To assess the binding selectivity of biotinylated p5+14 and p5R peptides, we used a commercial glycosaminoglycan (GAG) binding microarray (ZBiotech, CO, USA), which contains 16 subarrays each consisting of 47 differing GAGs (varying lengths, charges, and composition (Supplementary Figure S1)) plus internal controls. Increasing concentrations of each peptide were evaluated on the subarrays. The GAG arrays consisted of exemplar hyaluronic acid (HA), heparin, chondroitin sulfate A/C (CS-AC), chondroitin sulfate D (CS-D), dermatan sulfate (DS), heparan sulfate (HS with low to intermediate sulfation levels), heparan sulfate (HS with high sulfation levels), and keratin sulfate (KS). Background corrected binding of peptides p5+14 and p5R, at 2 µg/mL, to each GAG is shown in Figure 4A,B (additional concentrations are shown in Supplementary Figure S2). Except for a highly sulfated CS-D (GAG28), both peptides exhibited preferential binding to heparin and highly sulfated HS GAGs. Little to no binding to HA, CS-AC, CS-D (with singular exception), DS, and KS was observed. For heparin and highly sulfated HS, the binding intensity closely paralleled the total negative charge for each GAG (Figure 4C). The binding of peptides p5+14 and p5R to the array exhibited similar patterns in selectivity with a strong and significant linear correlation (Pearson *r* = 0.9436, *p* < 0.0001, R^2^ = 0.89) (Figure 4D).

**Figure 5:**
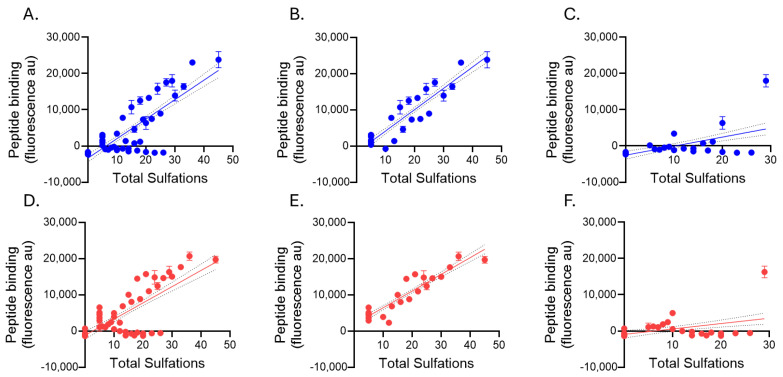
The binding of biotinylated peptides p5+14 and p5R to heparin and highly sulfated heparan sulfate is dependent on the total number of negatively charged sulfates. The interaction of p5+14 (**A**–**C**) and p5R (**D**–**F**) with GAGs correlated with the total sulfations on all GAGs, (**A**,**D**), total sulfations on heparin and HS (**B**,**E**), and total sulfations of non-heparin and HS GAGs (**C**,**F**). Linear regression analyses were performed and plotted with 95% confidence intervals. Peptides were tested at 2 µg/mL. The mean ± S.D. for each GAG panel is shown (*n* = 4).

Additional correlation analyses were performed to examine the relationships between the electrochemical characteristics of the GAGs (GAG length, total sulfation, and average sulfation per saccharide) and peptide binding. Significant, positive correlations were found between peptide binding—both p5+14 and p5R—and total GAG sulfations (Figure 5A,D and Table 2). The binding relationship was even greater (higher Pearson *r*) when the correlation was performed using just heparin and HS GAGs (Figure 5B,E). In contrast, very little binding was observed between the peptides and GAGs that were not heparin or HS (Figure 5C,F).

**Figure 6:**
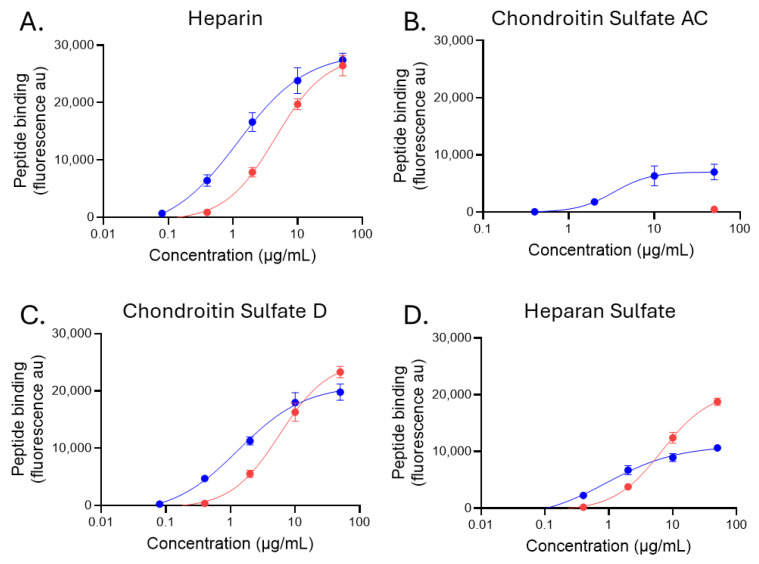
Binding potency of biotinylated peptides p5+14 and p5R with exemplar charged GAGs was greater for p5+14 compared with p5R. Binding (fluorescence au) of p5+14 (blue) and p5R (red) to select GAGs [(**A**) Highly sulfated heparin (GAG16); (**B**) CS-AC (GAG22); (**C**) CS-D (GAG28); (**D**) highly sulfated HS (GAG46)]. Data were analyzed using a sigmoidal 4PL curve fit to the data and an estimated apparent EC_50_ was determined. Mean ± S.D. are shown (*n* = 4).

One exemplar GAG from the groups of heparin, HS, CS-AC, and CS-D was chosen to assess the Ec_50_ (concentration at the midpoint of binding). Bound peptide fluorescence was plotted against the concentration of peptide and a sigmoidal 4PL binding curve fit to the data (Figure 6). For GAGs with high peptide binding (heparin, HS, and highly sulfated CS-D), Ec_50_ calculations could be performed for both biotinylated p5+14 and p5R (Figure 6A,C,D). However, p5R binding to CS-AC was at or below the background levels for all but one concentration (Figure 6B, red), and as such, we were unable to determine an Ec_50_ for p5R on that GAG (Table 3). In contrast, p5+14 exhibited saturable binding to the GAG, and an Ec_50_ was calculated (Figure 6B, blue; Table 3) despite the relatively low levels of binding when compared with heparin and HS (Figure 6A,D, blue). This analysis revealed that p5+14 bound with greater potency to the GAG substrates compared with p5R (Table 3). In this assay format, the Ec_50_ for the binding of biotinylated p5+14 to the highly charged heparin, HS, and CS-D ranged between 195 nM and 279 nM, whereas that of biotinylated p5R was characterized by a more than 5-fold lower potency, with Ec_50_ values between 1.3 and 1.95 μM. The differential binding potency, despite the shared selectivity, is likely due to the higher net charge on p5+14 (+12) compared with p5R (+8).

### 2.4. Heparan Sulfate Binding of Biotinylated Peptides p5+14 and p5R

**Figure 7:**
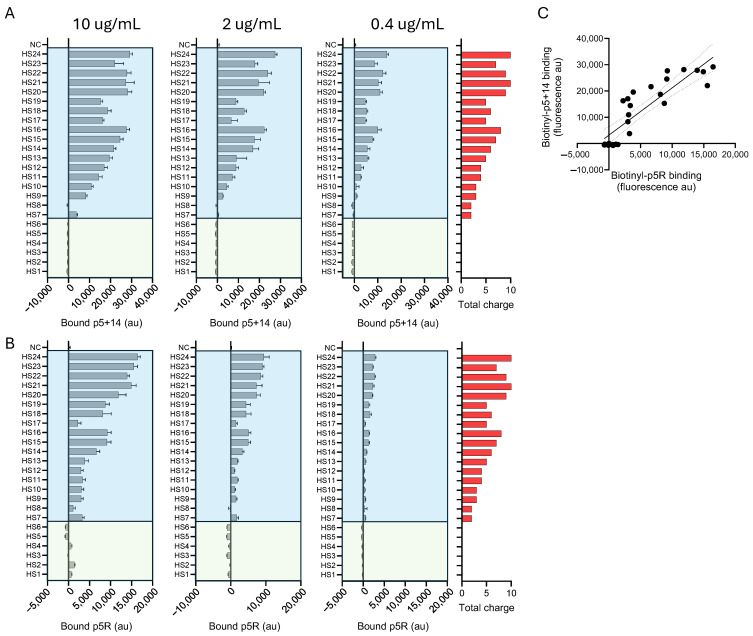
Heparan sulfate binding patterns of biotinylated peptides p5+14 and p5R. The binding of p5+14 (**A**) and p5R (**B**) was assessed on a commercial HS array at increasing concentration (0.4 µg/mL, 2 µg/mL 10 µg/mL). HS1–6 lacked sulfations with no net charge, whereas HS7–24 were of variable saccharide length and sulfation. The total charge on each GAG was calculated and plotted (red) for comparison. (**C**) Pearson’s correlation analysis for p5+14 and p5R binding to GAG was performed and the data fit to a linear regression and plotted with 95% confidence interval. The mean ± S.D. is shown in A and B (*n* = 6).

Since both peptide p5+14 and p5R exhibited selective binding to heparin and HS (Figure 4), we performed further studies using a specific HS microarray (ZBiotech, CO, USA). This array comprised 24 structurally unique synthetic HS domains that varied in length, net charge, and charge density (Supplementary Figure S3). The binding of biotinylated peptides p5+14 (Figure 7A) and p5R (Figure 7B) to the HS array was performed as above, using increasing concentrations of peptide. The intensity of binding for both peptides, based on the fluorescence intensity, was closely related to the total charge (total sulfations) of the ligand (Figure 7, shown in red). This was particularly evident for p5+14, where the binding to HS appeared to correlate precisely with the total charge of the HS.

**Figure 8:**
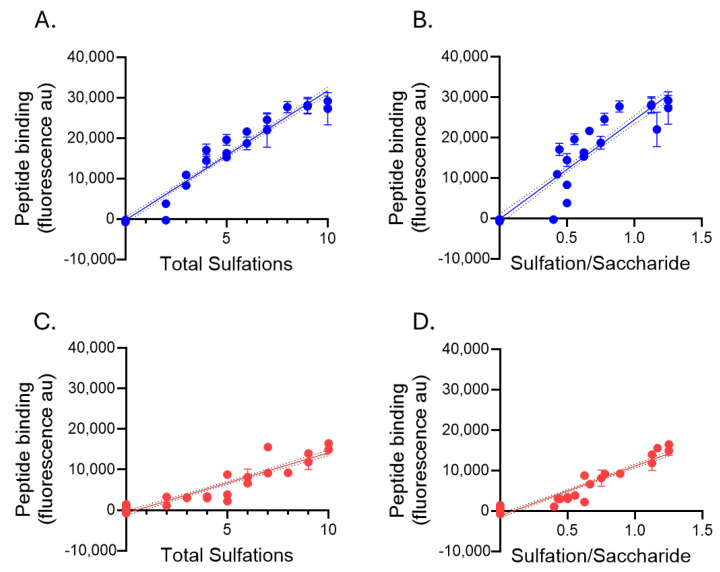
Biotinylated peptide p5+14 and p5R binding to HS ligands is dependent upon the charge and charge density. The total sulfations and the average sulfations per saccharide (charge density) were calculated, and the correlation between these characteristics and the binding of p5+14 (**A**,**B**) and p5R (**C**,**D**) were analyzed by linear regression. Mean ± S.D. is shown (*n* = 6).

At the risk of over-interpreting the data, it did appear that the interactions of peptide p5R with HS were more complex. For example, the binding of p5R to HS7–HS13 was approximately equivalent, whereas the net charge increased from +2 (HS7) to +5 (HS13). Then, the p5R reactivity doubled as the 6-O-sulfation increased, and the net charge increased to +6 and higher (HS14–HS16). Interestingly, HS17 (+5 charge) exhibited low activity with p5R, and the addition of just one more 2-O-sulfation on iduronic acid resulted in a 4-fold increase in binding (HS18). Despite potentially nuanced selectivity in peptide binding to HS, the interaction of biotinylated peptides p5+14 and p5R with HS exhibited a strong and significant linear correlation (Pearson *r =* 0.877, *p* < 0.0001, R^2^ = 0.7684; Figure 7C). The binding intensities of both biotinylated peptides to the HS array appeared to be dependent upon the net HS charge. We therefore explored the correlation between peptide binding to HS with total sulfations (net charge) and the number of sulfations per saccharide unit (a measure of charge density). Pearson’s correlations were performed to assess the linear relationship (Figure 8 and Table 4). For both p5+14 (Figure 8A,B) and p5R (Figure 8C,D), there was a strong (*r* > 0.9) linear and significant (*p* < 0.0001) correlation between the binding and charge-related parameters (Table 4).

**Figure 9:**
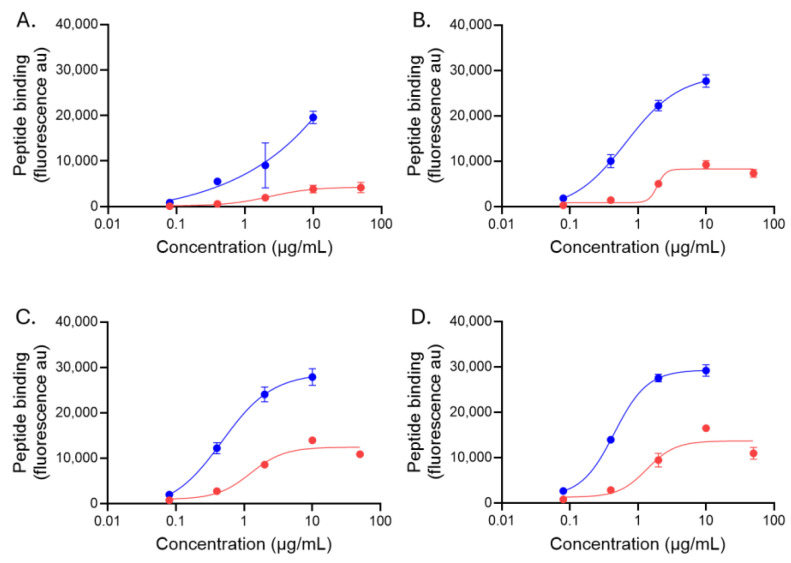
Biotinylated peptides p5+14 and p5R have different affinities for heparan sulfate glycans. Binding of p5+14 (blue) and p5R (red) to HS glycans ((**A**), HS13; (**B**), HS16; (**C**), HS22; and (**D**), HS24) was assessed at increasing peptide concentrations and the data fit to a sigmoidal 4PL curve and the apparent Ec_50_ calculated. Selected heparan sulfates are shown with specific binding curves. Mean ± S.D. is shown, *n* = 6.

Biotinylated peptide binding to four of the HS glycans in the array were analyzed further to assess the estimated binding potency (Figure 9). At equivalent mass, the binding of p5+14 to the HS13 glycan did not reach saturation; therefore, the binding potency could not be estimated (Ec_50_) (Figure 9A and Table 5). Peptide p5R did not bind to the same extent (based on the maximum fluorescence arbitrary units at saturation and interpolated binding potency [Ec_50_]) compared with p5+14 on any of the HS glycans tested (Figure 9B–D and Table 5). The HS glycans used in this binding analysis contained fewer saccharide units and less total charge compared with that shown in Figure 6D, which may explain the difference in the estimated potency data.

## 3. Discussion

Over the last 15 years, polybasic peptides with various structural and charge properties have been developed and studied for their ability to bind amyloid and serve as a vector for delivering radionuclides and biological agents to amyloid deposits [36,37,39,40,48,49,57,58,59]. This peptide family is characterized by its high net positive charge. Most are predicted to adopt an α-helical secondary structure based on the amino acid sequence; however, peptides with predicted β-sheet or random structures have also been identified as amyloid targeting agents [55]. All of the effective peptides comprise L-amino acids, with D-enantiomer variants losing the specific amyloid reactivity [60,61]. This peptide platform has been used to deliver radionuclides [36,37,39,40,41,42,43,46,47,48,49,50,51,53], immunoglobulins [59], immunoglobulin Fc domains [57,62], and peptide epitopes [58,63] to amyloid. The p5+14 and p5R peptides evaluated in this study are derived from the prototypic peptide, designated p5, and differ by the addition of 14 amino acids or by substituting arginine for lysine, respectively [40,52].

Amyloid-targeting peptides were originally identified based on our hypothesis that amyloid-associated HSPGs are highly charged relative to those found in healthy tissues [18,21,22] and could serve as a ligand for a pan-amyloid reactive peptide. As further evidence for hypersulfated HS in systemic amyloid deposits, the heparan binding single chain variable fragment (scFv), NS4F5, which binds to the highly sulfated HS motif (GlcNS6S-IdoA2S)3, was shown to specifically react with amyloid deposits in a murine AA model [64]. We therefore interrogated synthetic, heparin-binding peptides for their ability to specifically bind systemic AA amyloid in mice with no off-target reactivity [36,40]. We have since demonstrated that these amyloid-reactive peptides can, in addition to heparin, bind synthetic amyloid-like fibrils composed of light chain variable domains or the Aβ(1–40) peptide (in the absence of glycosaminoglycans), representing a second structural component of all amyloid deposits the peptides are capable of binding [38,55].

**Figure 10:**
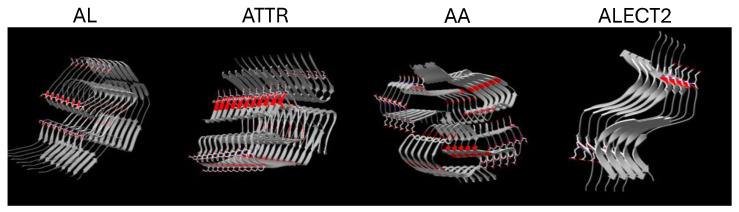
Predicted structure of human tissue amyloid fibrils. The structures of patient-derived amyloid fibrils, determined by cryo-electron microscopy, were rendered in DeepView PDBviewer using deposited PDB data. The acidic amino acids (aspartate and glutamate) are shown (red), and those potentially exposed on the putative fibril surface have side chain renderings. Images were generated from the following PDB structures: AL: 1BJM [66]; ATTR: 6SDZ [67]; ALECT2: 8G2V [68]; Human AA: 4IP8 [69].

Comprehending the interactions of the peptides with diverse types of amyloid fibrils is experimentally complex; however, we are currently performing molecular dynamic simulations and docking studies to explore the binding of peptide p5+14 with amyloid fibril structures available in the ProteinDataBank (Figure 10). Meanwhile, in this study, we explored the structural requirements of GAGs that favor the binding of peptides p5+14 and p5R. Both peptides bind GAGs with high levels of sulfation and a high charge density (Figure 4 and Figure 5). Peptide binding was greatest to heparin and HS as well as single highly sulfated CS-D species (generally a marine form of CS). Despite the high total charge of the CS GAG in the array, peptide binding was low, with a singular exception (Figure 4). This may be due to differences in charge density or spacing relative to HS, or the disaccharide composition (e.g., N-acetylgalactosamine in CS compared with N-acetylglucosamine in HS). What is remarkable is the abrupt intense reactivity of the peptides with GAG28, but none of the other CS GAGs (GAG23-GAG27). This suggests that, unlike HS where the reactivity increases linearly with the total charge, there is a minimum requirement for charge on CS-D, below which no reactivity is observed. This phenomenon has not yet been explored further.

In this assay format, biotinylated p5+14 exhibited a higher affinity for both heparin and HS immobilized in the arrays compared with biotinylated p5R (Table 3 and Table 5). This may be due to the extended nature of p5+14 (+12 charge) compared with p5R (+8 charge). These additional residues may enable enhanced binding despite lysine’s lower relative affinity compared with arginine [65]. The binding to HS species in the HS array further demonstrated that potent peptide reactivity correlated with both the total number of sulfations and the negative charge density (Figure 8). These selective interactions with HS species contribute to our explanation of pan-amyloid reactivity. We propose that peptides p5+14 and p5R share common modes of interaction with amyloid, in other words, the well-ordered array of basic amino acids in the peptides align with and bind the linear highly sulfated domains on the heparan sulfate glycans and the acidic side chains exposed along the long axis of amyloid fibrils (Figure 10).

Despite the fact that polybasic, pan-amyloid reactive peptides bind HS, the in vivo biodistribution is markedly distinct compared with a prototypical HS binding peptide such as basic fibroblast growth factor (bFGF). Negligible amyloid-reactive peptide binding was observed in healthy tissues compared with bFGF, which exhibited strong reactivity with healthy liver tissue and renal glomerulae [53]. These data further demonstrate the specificity of the pan-amyloid reactive peptides for systemic amyloid deposits through interactions with either fibrils or the unique amyloid-associated hypersulfated HS (or both). The amyloid specificity and pan-amyloid reactivity of these peptides have been further demonstrated in human patients through imaging studies of the radiolabeled peptide p5+14. This peptide serves as the basis of both an ^124^I- and ^99m^Tc-labeled radiotracer for imaging systemic amyloid deposits, including cardiac amyloid, by using PET/CT and SPECT/CT imaging, respectively [42,43,51]. Iodine-124 labeled p5+14 (iodine ^124^I-evuzamitide) is in a pivotal multicenter Phase 3 study for detecting cardiac amyloidosis. Peptide p5R is also clinically important and is the amyloid-binding moiety in the IgG1-peptide fusion *zamubafusp alpha*, which is being evaluated in a Phase 1 clinical study as an amyloid-clearing agent (NCT05521022). Considering the potential clinical utility of these peptides as components of novel pan-amyloid diagnostic and therapeutic agents, exploring their potential modes of amyloid binding is of great interest.

The concept of pan-amyloid reactive agents is not a new one. Small molecule dyes such as Congo red and thioflavin T bind all types of amyloid but not in a specific way (the former also binds cotton and the latter also binds DNA); nonetheless, a structural binding site is present that allows these reagents to interact with amyloid fibrils. The binding sites are recurring with defined periodicity, which results in Congo red birefringence when stained amyloid is viewed under cross polarized illumination [70],a and a red shift in the emission maximum and increase in the quantum yield when thioflavin T binds amyloid [71]. The interaction of thioflavin T with amyloid fibrils has been exploited to develop the Aβ amyloid imaging agent [^18^F]-flutemetamol [72,73]. Biological reagents that exhibit pan-amyloid reactivity include serum amyloid P-component [74] and the camelid antibody B10 [75]; the latter acts as a pattern recognition receptor that engages with the repeating charged elements of amyloid fibrils [76].

We hypothesize that the surface-catalyzed induction of peptide α-helicity serves to align the basic amino acid side chains along one face of the α-helix, allowing multivalent electrostatic interactions with the target ligand. We have conceptually likened this process to an electrostatic “zipper”, where the formation of electrostatic interactions between peptide residues and charged moieties on the target (acidic amino acid side chains or sulfate groups) induce helicity. Based on our structural studies using circular dichroism, both peptides p5+14 and p5R exist in partially unfolded states in solution (PBS pH 7.2) (Figure 2) [52,55]. However, in the presence of low molecular weight heparin (enoxaparin), as a surrogate for hypersulfated heparan sulfate, the peptides adopt an α-helical structure (Figure 2), supporting our electrostatic “zipper” hypothesis.

Peptides p5+14 and p5R are structurally similar, bind many types of amyloid, and may prove to be clinically invaluable components for diagnosing and treating systemic amyloidosis of any type. The studies described herein contribute to our model of target-catalyzed conformational changes in the peptides that result in amyloid binding. Future studies will include in silico analyses of the peptides with fibrils to add to our understanding of these interactions. We will also further exploit these peptides to deliver therapeutic payloads to tissue amyloid with the goal of providing a diverse armamentarium of treatment strategies for amyloid clearance.

## 4. Materials and Methods

### 4.1. Peptide Synthesis

Peptides were purchased from Genscript (Genscript Biotech, Piscataway, NJ, USA) as lyophilized products with >85% purity, resuspended in water, and stored at −20 °C. Peptides p5R and p5+14 were synthesized with and without biotin on their amine terminus.

### 4.2. Peptide Structure Modeling

Models of secondary structure were generated using the iTasser software package [77] and visualized in Deepview/Swiss-PdbViewer v 4.1.1 (Swiss Institute of Bioinformatics) [78].

### 4.3. Circular Dichroism

Peptide secondary structures were analyzed by circular dichroism using an Olis RSM 1000 (On-line Instrument Systems, Inc., Bogart, GA, USA). Each study consisted of three replicates that were averaged and the dilution/background spectra subtracted. Non-biotinylated peptides were diluted to 0.05 mg/mL in phosphate buffered saline (PBS). For experiments involving the addition of enoxaparin (Amphastar Pharmaceuticals Inc., Rancho Cucamonga, CA., USA), enoxaparin was added to diluted peptide in PBS to reach a final concentration of 0–66.7 µg/mL. For experiments involving the addition of 2,2,2-trifluoroethanol (TFE) (Thermo Fisher Scientific, Waltham, MA, USA), TFE was added to diluted peptide in PBS to reach a final concentration of 0–40%.

### 4.4. Glycosaminoglycan and Heparan Sulfate Arrays

Glycosaminoglycan (GAG) microarray binding studies were performed by Z Biotech (Z Biotech, LLC, Aurora, CO, USA). Briefly, microarrays were laid out in a 16-subarray arrangement with 47 GAG or 24 heparan sulfate (HS) binding targets per subarray. Spotted arrays were blocked at room temperature (RT) for 30 min with Glycan Array Blocking Buffer (Z Biotech, LLC, Aurora, CO, USA) before being washed. Biotinylated peptides were diluted in blocking buffer + 1% bovine serum albumin to final concentrations of 50, 10, 2, 0.4, and 0.08 µg/mL and the solutions added to the arrays for 1 h at RT. The arrays were then washed again and streptavidin-Cy3 (0.2 µg/mL) added, followed by a 1-h incubation. The fluorescence emission was quantified by scanning at 532 nm and evaluated using Mapix microarray software (Innopsys, Carbonne, France). Background control data were subtracted from binding signals before the data were averaged.

### 4.5. Radiolabeling of Peptide p5+14 and PET/CT Imaging

Patients were imaged with p5+14 as part of the first-in-human, open label, Phase 1 study (NCT03678259). Synthesis of ^124^I-labeled p5+14 has previously been reported [48]. Briefly, lyophilized human grade peptide (Attralus, Inc., Naples, FL, USA) was radiolabeled on the day of use with iodine-124 (3D Imaging, Little Rock, AR, USA) using iodogen as the oxidant. Subjects were administered a 2 mCi dose of I-124 (<2 mg of peptide) intravenously. PET/CT images were acquired from crown to thighs at 5 h post injection, using a Siemens Biograph PET/CT (Siemens Healthineers, Malvern, PA, USA) with 5 min bed positions for the PET and a low dose CT (120 kVp, 50 effective mAs) [42,48,51].

Data were reconstructed using a 3-dimensional ordered subset expectation maximization algorithm with attenuation and prompt gamma corrections, a 168 × 168 image matrix, and an image resolution of ~8 mm full width at half maximum. CT reconstruction utilized a medium smoothing kernel and 4 mm reconstruction increments. Patient PET/CT images were generated using Inveon Research Workplace software (Ed. 4.2 [4.2.0.15]; Siemens Preclinical Solutions). 

The study protocol was allowed to proceed by the U.S. Food and Drug Administration and performed under the auspices of Investigational New Drug (IND) submission No. 132282. Further approval was obtained from the Institutional Review Board at the UT Graduate School of Medicine (No. 4386). All patients provided written consent for the prescreening of medical records and the imaging protocol prior to participation in the study.

### 4.6. Data Visualization and Statistical Analysis

Data visualization, statistical analysis, and correlations were performed using GraphPad Prism v 9.4.1 or later. Correlations of peptide binding to microarray substrates were performed using Pearson’s correlations. Apparent affinities (Ec_50_) of peptides to array substrates were calculated using sigmoidal 4PL curve fitting to calculate the Ec_50_ in µg/mL, which was converted to molarity using the appropriate peptide molecular weight.

## Figures and Tables

**Table 1:** Physical characteristics of peptides p5+14 and p5R.

Peptide	Sequence	Molecular Weight ^1^	Amino Acid Length	Basic Residues	Average Charge per Residue
p5+14	GGGYS KAQKA QAKQA KQAQK AQKAQ AKQAK QAQKA QKAQA KQAKQ	4766.5	45	12	0.267
p5R	GGGYS RAQRA QARQA RQAQR AQRAQ ARQAR Q	3481.8	31	8	0.258

^1^ Molecular weight in Daltons.

**Table 2:** Pearson correlation analysis of biotinylated peptide binding to GAGs.

Biotinyl-Peptide		Sulfations (All GAGs)	Sulfations (heparin and HS Only)	Sulfations (Non-Heparin and HS)
p5+14	Pearson *r*	0.7925	0.9181	0.5309
	*p*-value	<0.0001	<0.0001	0.0063
p5R	Pearson *r*	0.7215	0.9223	0.3714
	*p*-value	<0.0001	<0.0001	0.0676

**Table 3:** Apparent binding potency of biotinylated peptides p5+14 and p5R to exemplar GAGs.

Apparent EC_50_: µg/mL (Molarity)				
Biotinyl-Peptide	Heparin (GAG16)	Chondroitin Sulfate AC (GAG22)	Chondroitin Sulfate D (GAG28)	Heparan Sulfate (GAG46)
p5+14	1.198 (251.3 nM)	3.376 (708.2 nM)	1.334 (279.9 nM)	0.932 (195.5 nM)
p5R	4.537 (1.303 µM)	nd *	5.758 (1.65 µM)	6.792 (1.95 µM)

* nd, not determined.

**Table 4:** Pearson correlation analysis of biotinylated peptide binding to HS.

Biotinyl-Peptide		HS Total Sulfations (Net Charge)	HS Sulfations/Saccharide (Charge Density)
p5+14	Pearson *r*	0.9764	0.9250
	*p*-values	<0.0001	<0.0001
p5R	Pearson *r*	0.924	0.9446
	*p*-values	<0.0001	<0.0001

**Table 5:** Apparent binding potency of biotinylated peptides p5+14 and p5R to exemplar HS glycans.

Apparent Ec_50_: µg/mL (Molarity)				
Biotinyl-Peptide	HS13	HS16	HS22	HS24
p5+14	-	0.6685 (140.2 nM)	0.4734 (99.31 nM)	0.4482 (94.03 nM)
p5R	2.338 (671.5 nM)	1.930 (554.3 nM)	1.241 (356.4 nM)	1.358 (390.0 nM)

## Data Availability

The original contributions presented in this study are included in the article/Supplementary Material. Further inquiries can be directed to the corresponding author.

## Supplementary Materials

The following supporting information can be downloaded at: https://www.mdpi.com/article/10.3390/ph18091340/s1. Figure S1: Glycosaminoglycan array structures in Symbol Nomenclature for Glycans (SNFG) format, Figure S2: Glycosaminoglycan array peptide titrations, Figure S3: Heparan sulfate array structures in Symbol Nomenclature for Glycans (SNFG) format.
