# Supplementary material for: Pan-Amyloid Reactive Peptides p5+14 and p5R Exhibit Specific Charge-Dependent Binding to Glycosaminoglycans
Source: Pharmaceuticals (Basel). 2025 Sep 6;18(9):1340. doi: 10.3390/ph18091340 (PMC12472597; doi:10.3390/ph18091340)
Supplement: Supplementary file 1 [file pharmaceuticals-18-01340-s001.zip › pharmaceuticals-3783093-supplementary.pdf]

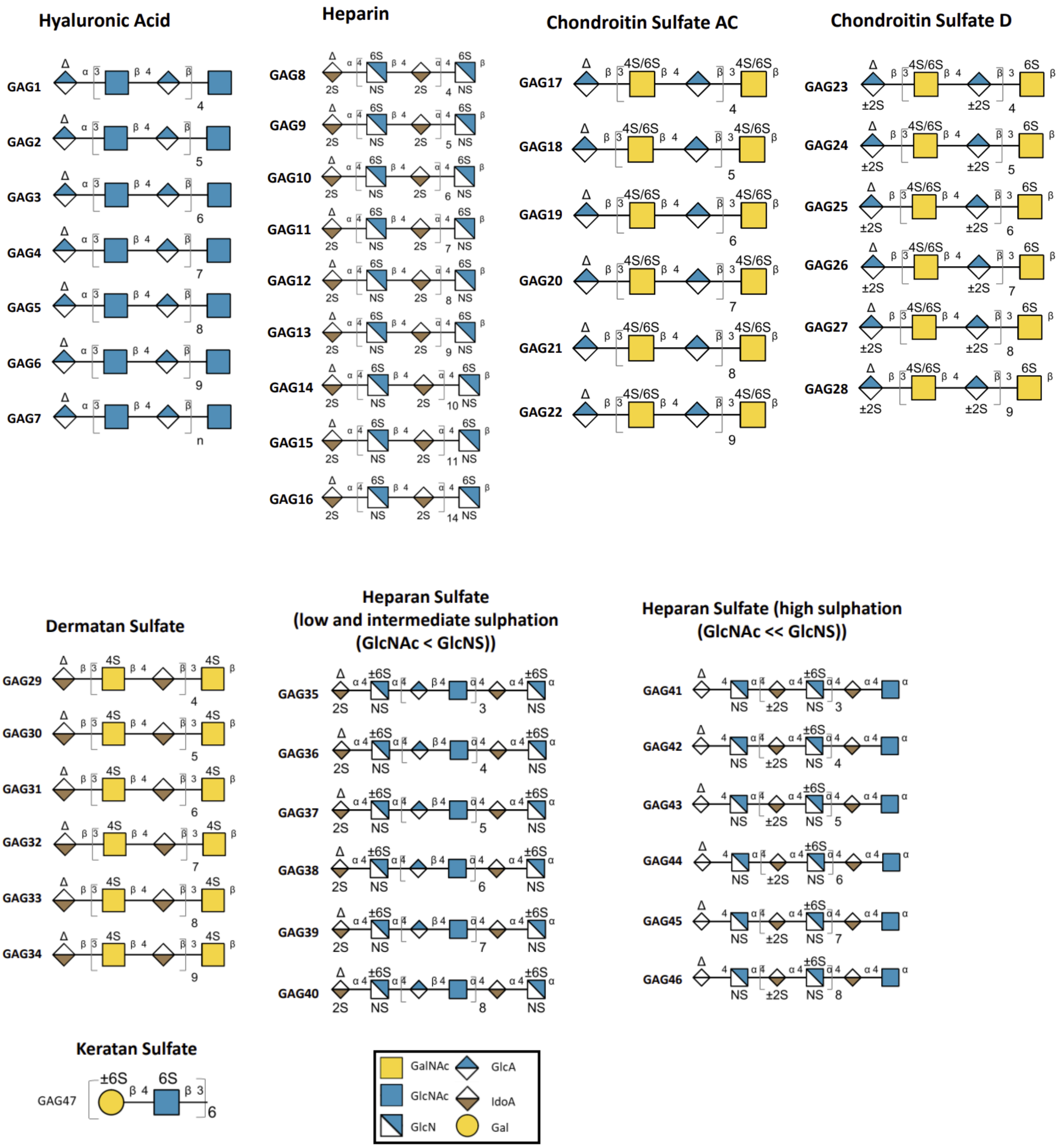

Figure S1: Glycosaminoglycan array structures in Symbol Nomenclature for Glycans (SNFG) format.

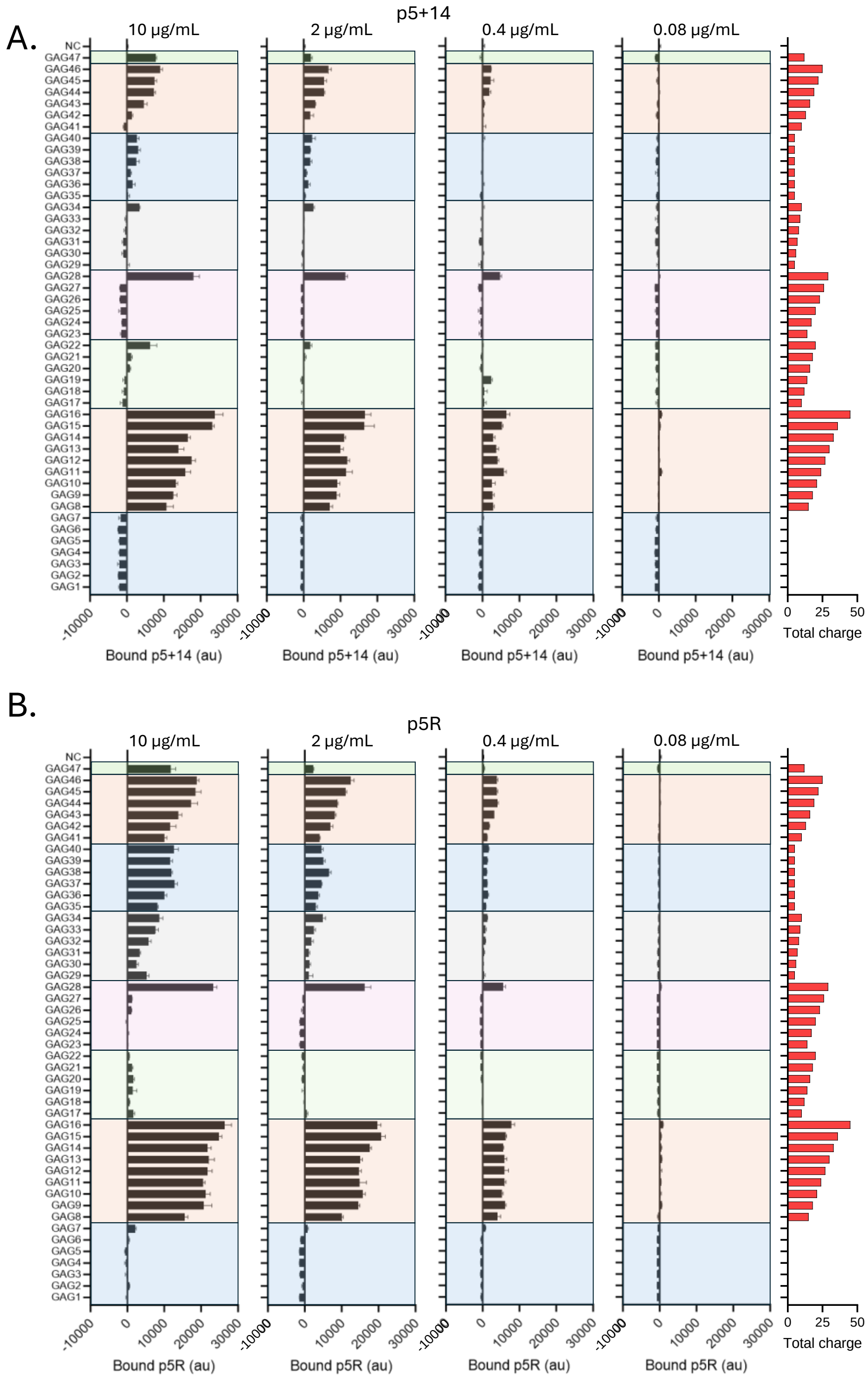

Figure S2: Glycosaminoglycan array peptide titrations.

Peptides p5+14 (A) and p5R (B) were tested on a GAG microarray at four concentrations (10 µg/mL, 2 µg/mL, 0.4 µg/mL, and 0.08 µg/mL). Chart in red represents the total number of sulfations per glycosaminoglycan tested. Mean ± S.D. shown for each GAG.

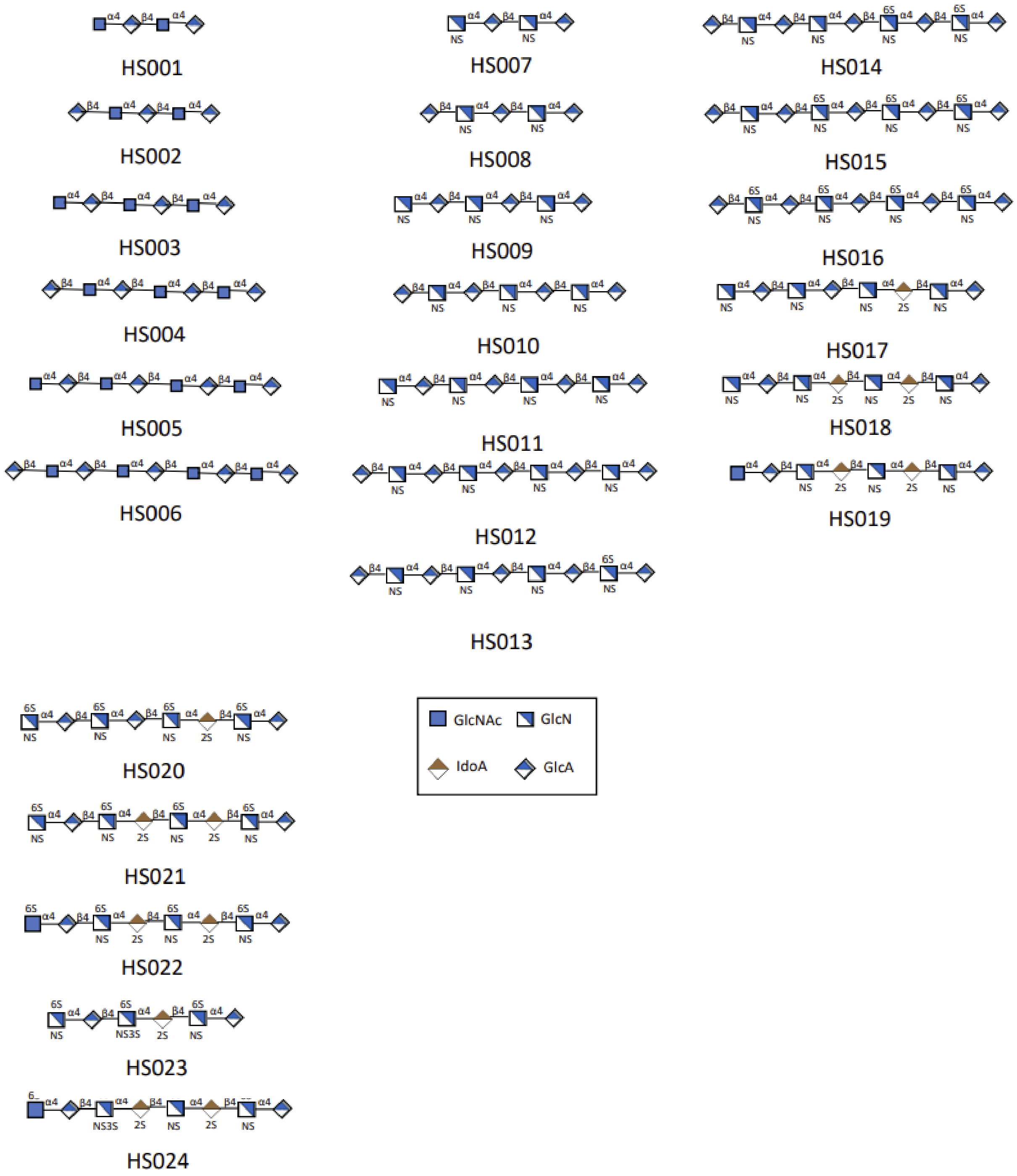

Figure S3: Heparan sulfate array structures in Symbol Nomenclature for Glycans (SNFG) format.
